# Effect of a Short-Term Intervention with *Lactobacillus salivarius* Probiotic on Early Childhood Caries—An Open Label Randomized Controlled Trial

**DOI:** 10.3390/ijerph191912447

**Published:** 2022-09-29

**Authors:** Małgorzata Staszczyk, Małgorzata Jamka-Kasprzyk, Dorota Kościelniak, Beata Cienkosz-Stepańczak, Wirginia Krzyściak, Anna Jurczak

**Affiliations:** 1 Department of Pediatric Dentistry, Institute of Dentistry, Jagiellonian University Medical College, 31-155 Krakow, Poland; 2 Laboratory of Anthropology, Institute of Zoology and Biomedical Research, Jagiellonian University, 30-387 Krakow, Poland; 3 Department of Medical Diagnostics, Faculty of Pharmacy, Jagiellonian University Medical College, 30-688 Krakow, Poland

**Keywords:** pediatric dentistry, early childhood caries, dental public health, caries prevention, probiotics, *Lactobacillus salivarius*

## Abstract

ECC is a significant therapeutic and social problem and a global burden on public health. The aim of this clinical trial was to test whether a 2-week daily consumption of chewing tablets containing thermally inactivated *L. salivarius* reduces the 12-month caries increment compared to the control group. The investigation was a single-center, randomized, controlled open-label, blinded end-point evaluation trial in two parallel groups. At baseline, 140 generally healthy children between 3 and 6 years of age with or without ECC were randomly assigned to the probiotic test group (*n* = 70) or to the treatment as the usual control group (*n* = 70). The primary outcome measure was the 1-year increment in caries incidence and prevalence. Secondary endpoints assessed were the initial, cavitated and obvious dentinal caries increment as well as the measurement of dental plaque accumulation, as an indicator of the ECC risk. Data were collected through the clinical assessment of the children’s caries (dmft and ICDAS II) and oral hygiene status (DI-S of OHI-S index). Caries incidence and prevalence were statistically significantly lower in the probiotic group versus the control group (*p* < 0.001 and *p* = 0.0075). The initial and final mean OHI-S scores in the probiotic group did not show any significant differences. In conclusion, the regular short-term intake of probiotics may reduce caries development. Our findings suggest that self-administered probiotic therapy may provide a good complement to increase the effectiveness of individual preventive home care in preschool children. This is the first clinical study evaluating the effect of a short-term probiotic intervention on reducing early childhood caries with 12 months of follow-up.

## 1. Introduction

Oral health is an integral part of general health. WHO data indicate that early childhood caries (ECC), defined as the presence of one or more caries teeth removed or filled in children under the age of 6, is one of the most common diseases worldwide among children of this age [1]. It affects over 600 million children worldwide and despite the implemented preventive and health education programs, it remains a global burden on public health with a wide variation in prevalence and severity across countries with different levels of income [2]. The incidence of ECC ranges from 23% to 90%, with most countries exceeding 50% [3,4]. In Poland, the problem is non-decreasing and it is found in over 76% of preschool children, including about 54% of 3-year-olds [5].

The first signs of ECC, despite dental interventions, pose a high risk of disease progression and may cause pain and inflammatory complications in a short time, finally affecting overall quality of life and generating health and economic burdens [6,7,8,9]. ECC is, therefore, a significant therapeutic and social problem, and it has caused researchers to focus on the complex, multifactorial pathomechanism of the disease, which is a classic example of mutual interactions between the external environment, microorganisms and host susceptibility [10]. The development of caries is determined by an imbalance between pathological factors leading to the demineralization of tooth tissue (acid-producing bacteria, impaired saliva function or frequent consumption of fermentable carbohydrates) and protective factors causing remineralization (the normal flow of saliva with its components, antimicrobial agents, the use of fluoride toothpastes or selected foodstuffs) [11]. Chewing gums that stimulate saliva flow and contain calcium or/and polyols can remineralize early caries lesions and significantly reduce the amount of *S. mutans* and *Lactobacilli* in the oral cavity, and so it has promising potential in the prevention of caries [12].

The pillars of caries prevention are: proper nutrition, oral hygiene and use of prophylactic agents containing fluorides [13,14,15]. In addition to these established procedures, there is a need for supplementary caries prevention tools, especially in the youngest age groups, in order to maintain symbiotic homeostasis of the oral microbiome (primary prevention) or restore it (secondary prevention).

The use of probiotics is a part of the concept of searching for potential new agents for caries prophylaxis by inhibiting the development and metabolism of cariogenic biofilms, as well as by increasing the growth and survival of microflora associated with oral health [16,17,18].

Additional benefits, such as improved general health and a reduced need for antibiotics in preschool children are also important [19,20]. According to the definition by the FAO/WHO (Food and Agriculture Organization of the United Nations, World Health Organization), probiotics are live microorganisms that do not cause negative consequences for the body and provide health benefits when administered in adequate amounts [21]. Recently, studies have given hope for the possibility of using probiotics in the prevention of caries but no type has yet been described in the literature as being most effective. Currently, probiotic strains originally serving to modulate the gastrointestinal microflora (the most common: the genera *Lactobacillus* and *Bifidobacterium*), are mainly used in the prevention of oral diseases [22,23,24].

The mechanism of action is not entirely clear as far as caries prevention with probiotics is concerned [25]. It is known that they can modulate the human oral microflora by competing with other bacteria for nutrients and substrate binding sites, inhibiting their growth by producing antimicrobial agents, and stimulating the body’s immune response [26,27]. This confirms their potential usefulness in modulating the oral microbial ecology in terms of caries prevention. Despite the current growing interest in the use of probiotics for caries prevention, their clinical efficacy in this aspect appears limited and controversial. Although in vitro studies have yielded promising results, clinical trials have not shown clear effects [28,29,30,31,32,33,34,35].

Existing systematic reviews and meta-analyses on this topic that include a change in microbiological parameters as an endpoint, showed a significant decrease in *S. mutans* after the use of different probiotic strains [36,37,38,39,40]. In contrast, a non-significant statistical reduction in SM levels was shown in systematic reviews by Twetman and Twetman-Keller [41,42].

Due to the limitations associated with the microbiological parameter (dysbiosis caused by many factors), studies that evaluate the effect of probiotics on changes in caries indices provide a more binding and direct answer to the question of the efficacy of probiotic strains in the prevention of ECC [43,44]. The results of the published clinical studies evaluating this issue range from no effect to a significant reduction in caries, with most being characterized by considerable heterogeneity and low-quality evidence. The results of several studies considering these parameters showed a significant reduction in caries severity in preschool children [19,20,28,29,30,45,46]. However, some literature data indicate that there were no statistically significant differences in the increment of caries between the study group and the placebo group [31,32,34]. The only meta-analysis on this topic that included changes in caries parameters as an endpoint did show a reduction in caries risk and incidence, but these changes were not statistically significant [39]. Systematic reviews, however, unanimously show promising results with clinical caries indicators, suggesting a positive effect of probiotics in caries prevention [37,41,42,47].

Furthermore, it is important to emphasize the scarcity of published clinical studies evaluating the efficacy of probiotics in the prevention and control of ECC [38]. There is also a lack of literature reports on this topic in relation to the *Lactobacillus salivarius* strain. The only existing studies showed a significant reduction in salivary *S. mutans* levels immediately after short-term supplementation with a probiotic containing this strain in 6-year-old children or in adults, but did not address the effect on caries parameters [48,49].

Due to the lack of adequate clinical evidence, probiotics, although promising for the prevention of dental caries, have not received clinical recommendations [21].

A preliminary in vitro study of the probiotic strain *Lactobacillus salivarius* (HM6 Paradens) noted that this probiotic inhibited the ability of *S. mutans* and *C. albicans* strains derived from the dental plaque of children with ECC, to form a common structure reducing the number of colonies grown and the biomass of the biofilm [50].

These promising results provided the rationale for including the preparation containing *L. salivarius* in our clinical trials.

The null hypothesis of the study was that supplementation with *Lactobacillus salivarius* (HM6 Paradens) does not reduce caries growth in children under six years of age. The primary objective was to compare changes in caries rates in the deciduous dentition of preschool children who received a two-week daily oral supplementation of *Lactobacillus salivarius* in the form of lozenges, with a group of children not taking the probiotic, taking into account the persistence of the effect obtained after supplementation (at 12-month post-supplementation). An additional aim was to compare children from both groups in terms of changes in hygiene index scores, i.e., the presence of visible plaque as an indicator of ECC risk, the biofilm of which is a major factor in caries development [10,11].

The study assessed whether the intake of a probiotic containing *Lactobacillus salivarius* reduces caries growth and caries risk in preschool children and whether it can be useful for individual, home-based ECC risk prevention and control as an adjunct method supporting existing fluoride prophylaxis methods. Any potential side effects of the probiotic were also observed.

## 2. Materials and Methods

### 2.1. Study Protocol

The prospective investigation was a single-center, randomized, controlled open-label, blinded end-point evaluation trial in two parallel groups [51].

The study was conducted at the Department of Pediatric Dentistry, Institute of Dentistry, Jagiellonian University Medical College in Kraków, Poland, according to the guidelines outlined in the Helsinki Declaration of 2013 [52], and followed the Consolidated Standards of Reporting Trials (CONSORT).

The design of the clinical trial, was approved by the Bioethics Committee of the Jagiellonian University (Kraków, Poland) (consent no. 1072.6120.31.2018).

### 2.2. Participants

#### 2.2.1. Selection Criteria

The study included healthy children (based on parents reporting on their children’s medical history) of either sex between 3 and 6 years of age with or without ECC. Other inclusion criteria were children who had not consumed antibiotics or probiotics in any form for three months prior to the study, children who were willing to chew the tablets and participate in the study, and parents of the subjects who were willing to give written informed consent and follow study procedures.

Exclusion criteria included children with severe infections, systemic diseases, weakened immune systems, congenital abnormalities, and food allergies as well as with inflammatory oral diseases, other oral diseases and periodontal pathology, and those who had installed an orthodontic correction device. The use of endogenic fluoride therapy, antibiotics, anti-inflammatory drugs or corticosteroids and a diet rich in probiotic products and supplements, such as vitamins or probiotics, within three months of the study initiation were also criteria for exclusion, along with partial or complete rejection of the dental examination by the child or their legal guardian and those not willing to participate in the study.

The purpose and procedure of the study were explained to all the subjects, and they were further informed that their refusal to participate would not disadvantage them in any way and that they were free to withdraw from the study at any time before they were asked to voluntarily sign the consent form.

#### 2.2.2. Settings and Locations Where the Data Were Collected

All participants were the patients of the University Dental Clinic in Kraków (Poland) and were screened/ recruited by researchers from the Department of Pediatric Dentistry, Institute of Dentistry, Jagiellonian University Medical College in Kraków.

According to epidemiological data that showed caries prevalence in 5-year-old children at the level of 74.9, the children from the Lesser Poland region represent the group with a high risk of dental caries, which is in keeping with the national rate of children with ECC in the Polish population (in Poland, it is found in over 76% of preschool children, including approximately 54% of 3-year-olds) [5,53].

The main sources of fluoride exposure to participants were tooth brushing with toothpaste containing 1000 ppm of fluoride. This region of Poland, as well as the rest of the country, is not engaged in any artificial tap water fluoridation program. The average content of fluoride in the drinking water in Kraków is below WHO recommendations (0.5–1.0 mg/L), and amounts to about 0.1 mg/L.

Oral health care of all of the children in our study was provided within the public health service through the University Dental Clinic, where the trial was performed.

### 2.3. Interventions

For ethical reasons, all the children participating in this study, both in the test and control groups, as patients of the University Dental Clinic in Kraków were routinely checked, and if necessary, dental treatment and professional caries prophylaxis were provided, depending on the level of risk of this disease and in accordance with the recommendations as an element of “usual care” [13,14]. Prior to the initiation of the study, in connection with the enrollment, all legal guardians of selected children received instructions on oral hygiene. They were instructed on how to brush with the appropriate amount (smear layer/rice or pea-size, according to the child’s age) of fluoridated toothpaste (1000 ppm) twice a day (morning and evening) during the entire study period, which is the basis of home caries prophylaxis, also constituting an element of “usual care”. Equal manual toothbrushes and toothpastes containing 1000 ppm of fluoride were given to the participants (from both groups) by the investigators.

Additionally, the children from the test group received regularly available for sale chewing tablets containing 10 mg thermally inactivated *Lactobacillus salivarius* HM-6 Paradens as well isomalt, sucralose, natural strawberry flavor, magnesium stearate and xylitol (200 mg/1 tablet; Acidolac Dentifix Kids; Polpharma, Poland). The guardians were instructed to give their child two tablets per day: 1 tablet in the morning (after breakfast) and 1 tablet before bedtime, always after brushing their teeth. After the intake, water or food ingestion was not allowed for two hours.

The parents were given tablets for 14 days, according to the design of the clinical trial; ClinicalTrials.gov identifier (NCT number): 02752594. They were asked to bring back to the clinical team all non-used tablets and filled questionnaires where they marked each tablet taken (+) by the child or missed (−) in the morning and evening. Compliance with the protocol was additionally evaluated and reinforced with children by the use of sticker charts (one taken tablet = one sticker applied). When ≤2 tablets per week were forgotten, the compliance was rated as “acceptable” and if this happened more frequently as “questionable”. The parents were asked to immediately report any possible harmful effects to the clinical staff and stop the intake, as well as any sickness or any other circumstances.

The control group was not administered any placebo chewable tablets for the same length of time, but other hygienic and dietary recommendations were the same in both groups, in line with the existing guidelines [13,14,15]. The guardians were asked to brush their children’s teeth twice a day, in the morning (after breakfast) and before bedtime and not allow children to eat any food within 2 h after toothbrushing.

### 2.4. Outcome Measures

The primary outcome measure of the present study was an increase in caries incidence/in decayed, missing due to decay, or filled primary teeth within the groups of preschool children between baseline and 1-year follow-up. An increase in caries prevalence/in participants who developed new caries lesions within the groups of preschool children between baseline and 1-year follow-up was also the primary efficacy parameter studied.

Secondary outcomes assessed were the initial, cavitated and obvious dentinal caries increment as well as the measurement of dental plaque accumulation on the teeth surfaces/presence of dental plaque within the groups of preschool children between baseline and 1-year follow-up.

### 2.5. Clinical Examination and Data Collection

The clinical examination consisted of an assessment of the children’s caries status and oral hygiene status (determination of dental plaque accumulation). The examination of all the participants was performed in a dental chair with an operating light using a dental mirror, air syringe, WHO periodontal probe, at the Department of Pediatric Dentistry, University Dental Clinic in Kraków. The patients were not exposed to radiographs. The visual and tactile detection of caries lesions in primary dentition was performed, after cleaning and drying tooth surfaces, in accordance with the guidelines of the World Health Organization for epidemiological studies on oral health [52].

Caries incidence at each examination was determined as the number of decayed, missing due to decay, or filled primary teeth (dmft), according to WHO oral health guidelines [52].

Caries prevalence at each examination was determined as the proportion of individuals with caries, being classified as affected (dmft > 0, ICDAS II 1–6 > 0).

The International Caries Detection and Assessment System II (ICDAS II classification) was used for describing the severity of caries lesions [54,55]. Code 0 corresponds to no caries lesions. Decayed teeth, used to measure (total) caries increment, were those coded as ICDAS II level 1 (first visual change in enamel seen after prolonged air drying) through 6 (distinct cavity with extensive visible dentine). Teeth that met the ICDAS II criteria for levels 1–2, 3–6 and 4–6 were separately considered as independent groups in order to show the caries increment for initial (early lesions limited to enamel in the form of opacity), cavitated and obvious dentinal caries, respectively, and to simulate what the increment would have been in terms of WHO criteria.

Clinical assessment of caries (dmft and ICDAS II) in each child, from both groups, with no distinction between whether the participant was in the experimental group or the control group, was performed at 2 identical time points; immediately before starting supplementation (baseline) and 12 months after its completion (single follow-up examination 1 year later).

Increment in caries incidence (Δ dmft, mean ± standard deviation) and in caries prevalence (Δ% children) was calculated as the difference between the follow-up and baseline scores.

Before dental assessment, the presence of visible plaque accumulation was scored using the Oral Debris component (DI-S-debris index) of the Simplified Oral Hygiene Index (OHI-S) of Greene and Vermillion based on numerical determinations representing the amount of debris or calculus on index tooth surfaces, after plaque disclosing [56]. The assessment of the OHI-S index, in each child from both groups, was performed at 4 identical time points. The pre-intervention plaque index (as the baseline data) was recorded one week after the complete oral prophylaxis performed for both groups (to ensure the homogeneity of the oral conditions of the subjects). The post-intervention plaque index was recorded again just after 2 weeks of regular administration of the probiotics with no distinction between whether the participant was in the experimental group or in the control group and statistically compared with the baseline data. Additionally, the OHI-S index was recorded also after 3 and 12 months from the end of probiotic supplementation and statistically compared with the baseline data, in order to assess the durability of the possible obtained effect.

All children were examined by two dentists, pediatric dentistry specialists (“gold standard”), who work in a primarily research-based clinic and have been trained specifically for the study examination. To minimize inter-examiner variability in data collection, including classification of caries lesions and recording of existing restorations, the dentists were calibrated against each other in dmft (decayed, missing, filled teeth) charting and to differentiate between sound surfaces, non-cavitated and cavitated caries lesions (ICDAS scores).

In the pilot study, 20 randomly selected 4-year-old children were examined independently by these two clinicians. The results have shown very good inter-examiner reliability in dmft and ICDAS scores (Kappa Fleiss values 0.90 and 0.92, respectively). Very good intra-examiner agreements were obtained by the two individual examiners for dmft scores (Kappa Fleiss values 0.94 and 0.83) and for ICDAS scores (Kappa Fleiss values 0.93 and 0.86, respectively). A reliability assessment of diagnosing the presence of dental plaque (OHI-S scores) resulted in very good intra-examiner agreement with the Kappa Fleiss coefficient values of 0.77 and 0.89, respectively for each of individual researchers, as well as in very good inter-examiner agreement with the Kappa Fleiss coefficient value of 0.87 [57].

### 2.6. Sample Size

The sample size of the participants was determined based on epidemiological data from the previous study in preschool children from the same region (mean [±standard deviation] dmft, 4.55 ± 4.16) [5,53]. It was estimated that 68 children needed to be allocated to each arm in order to detect a mean clinical reduction of 2.0 ± 4.16 (dmft) (α = 0.05 and β = 0.20), according to the Statistica program. The estimated proportion of dropouts was determined to be 10% per year.

### 2.7. Randomization, Allocation Concealment Mechanism, Implementation and Blinding

The enrollment of the children who complied with the eligibility criteria was conducted by one of the investigators after obtaining a signed, written informed consent from the legal guardians.

One of the authors excluded from subsequent stages of the study, including analysis of the results, generated the random 1:1 allocation sequence using the IBM SPSS Statistics (version 27.0, Armonk, NY, USA) programming with the aid of computer-generated numbers (Excel randomization tool). Using random numbers, the same researcher assigned the participants to one of two parallel groups, the probiotic group or to the control group. The participants were allocated 0 or 1 code in order to conceal their identity. The code was kept by the same author in sealed envelopes to ensure allocation concealment, and was not unveiled until all data had been analyzed.

The envelopes with the assignment of the probiotic or control treatments were provided to the clinical site. Additionally, other sealed bubble envelopes containing packets with or without tablets, but with similar weight, marked with number codes, were used to keep the study blinded to the researchers caring for participants, assessing health outcomes, analyzing data, as well as to the healthcare personnel of clinic. They were prepared and sent by the same author that generated the random allocation sequence to the clinic and then the investigators in turn distributed them to patients based on the randomization numbers.

### 2.8. Statistical Methods

The Shapiro-Wilk test indicated that the data did not conform to a normal distribution (*p* < 0.5). So, the difference between control and probiotic groups of children were analyzed using the Chi-square test for categorized/dichotomized variables and the nonparametric Mann-Whitney U test for interval variables. The nonparametric Friedman test was also performed to compare OHI-S scores at each time point for both groups. Post hoc comparisons were adjusted using the Durbin-Conover method. Multivariable logistic regression was used to analyze the risk of initial, cavitated and obvious lesions and supposed confounding variables (age, sex and control/probiotic group). Data were analyzed using IBM SPSS Statistics (version 27.0) and Statistica (TIBCO Software Inc., 2017, version 13, Palo Alto, CA, USA). The differences were considered as significant when *p* < 0.05.

## 3. Results

### 3.1. Participants Flow

At the start of the study, 286 children were recruited. The 146 pediatric subjects were excluded because they did not meet the selection criteria, they or their legal guardians refused to participate, or due to other reasons. Therefore, at baseline, there were 140 participants, all of whom were given informed consent by their legal guardians. The remaining subjects were randomly assigned to the probiotic test group (*n* = 70) or to the control group (*n* = 70).

**Figure 1:**
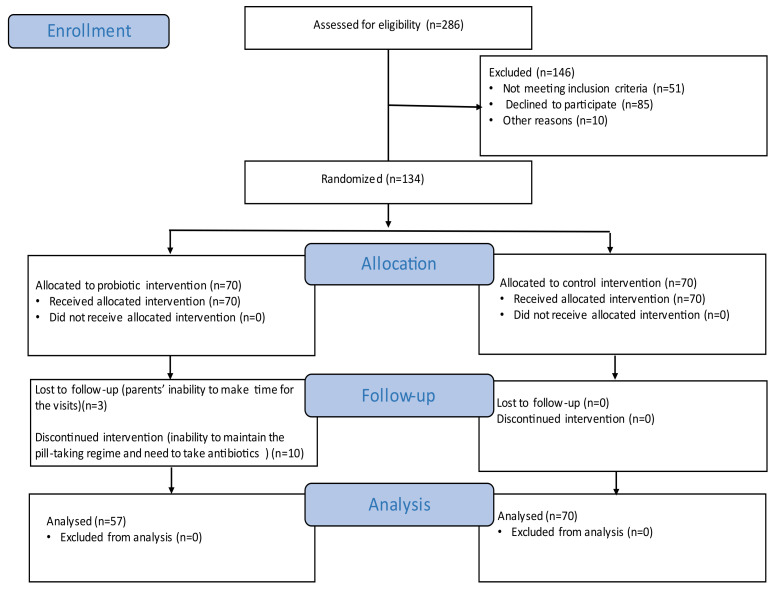
Research flow diagram (according to CONSORT).

During the study, 13 children dropped out from the probiotic group, since 10 of them discontinued the treatment (seven subjects dropped out due to their inability to maintain the pill-taking regime and three subjects due to their need to take antibiotics) and three were lost to follow-up (due to their guardians inability to make time for the visits). There were no dropouts from the control group during the study; therefore, all 70 children completed the study. The final participant count for both groups was 127, and their data were used to analyze the efficacy endpoints (Figure 1).

### 3.2. Recruitment

The enrollment of children started in September 2018 and the final recordings were conducted in March 2021.

### 3.3. Baseline Data

The baseline general, demographic and clinical characteristics of the children in the intervention group and the control group were compared to confirm the homogeneity of the two groups (Table 1). There were no statistically significant differences between the groups in age and gender distribution, at the baseline (*p* = 0.76 and *p* = 0.88, respectively).

The number of children with caries lesions in total (ICDAS II 1–6 > 0) was 50% in the control and 49.12% in the probiotic group, respectively, showing no significant difference between the two groups (*p* = 0.93). There were no significant differences between the groups in the mean total caries incidence; mean number of new decayed, missing due to decay or filled primary teeth (dICDAS 1–6 mft) (*p* = 0.83).

At baseline, the groups were not balanced only according to the mean OHI-S scores (*p* = 0.04). All children who remained in the study did not differ significantly from those who dropped out (examine groups vs dropouts). As all the dropouts were from the probiotic group, the children from the probiotic group who continued the study and those who dropped out were compared and there were no significant differences between them (probiotic vs dropouts).

### 3.4. Numbers Analyzed

In the recommended per protocol analysis group test, the data of one hundred twenty-seven children who participated in the end-of-trial visit were assessed.

### 3.5. Effect on Dental Caries

Caries data at baseline and at the end of the study are shown in Table 2 and Table 3. The caries incidence in the children of the intervention group and the control group was balanced at baseline in terms of the level of non-cavitated initial (dICDAS 1–2 mft) (*p* = 0.661), cavitated needed to treat (dICDAS 3–6 mft) (*p* = 0.865) and obvious dentinal caries lesions (dICDAS 4–6 mft) (*p* = 0.931) [55]. In addition, there were no significant differences in the number of children with initial (ICDAS 1–2 > 0), cavitated (ICDAS 3–6 > 0) and obvious dentinal (ICDAS 4–6 > 0) caries lesions between both groups (*p* = 0.94, *p* = 0.60 and *p* = 0.12, respectively) (Table 2).

At the end of the study the increment of new decayed, missing due to decay, or filled primary teeth within the groups (expressed as ΔdICDAS 1–6 mft, 12 months—baseline) was statistically significantly lower in the probiotic group versus the control group (*p* < 0.001) with the large effect size (d = 1.087) (Table 2).

The 1-year non-cavitated caries increment (ΔdICDAS 1–2 mft) and cavitated caries increment (ΔdICDAS 3–6 mft) were significantly lower in the probiotic group compared with the control group; (*p* = 0.002 and *p* < 0.001, respectively), both with intermediate effect size. When examined at the obvious dentinal caries lesion level (ΔdICDAS 4–6 mft) after the 12-month follow-up period, the probiotic group exhibited a significantly lower increment versus the control group (*p* < 0.001) with intermediate effect size (Table 2).

The final increment in caries prevalence in the probiotic group was found significantly lower (*p* = 0.0075) relative to the control group with odds ratio (OR) of 0.0958 (*p* = 0.027) and the prevented fraction was 90%. Based on the caries prevalence, the absolute risk reduction (ARR) was 13.96% (95% CI 4.78–23.14%) and the number needed to treat was 7.2 (95% CI 95% 4.3–20.9) (Table 3). A statistical significance (*p* = 0.0331) in the relative risk values (RR) of 0.1116 (95% CI 0.0149, 0.8391) for children with total caries lesions (ICDAS 1–6 > 0), indicated association between the consumption of probiotic tablets and the appearance of new caries lesions (Table 3).

Probiotic tablets intake showed also a statistically significant OR of 0.2520 (*p* = 0.0037) and of 0.0302 (*p* = 0.0008) for participants who developed new ICDAS 3–6 and ICDAS 4–6 lesions, respectively, indicating that individuals from the probiotic group have a significantly lower probability of manifesting caries increment (cavitated and obvious dentinal lesions, respectively) during the follow-up period than children from control group (Table 3), that was confirmed after logistic regression (multivariate analysis) (Table 4 and Table 5). The number of participants who finally developed new initial caries lesions (ICDAS 1–2 > 0) was higher in the control group than in the probiotic group but the difference was not statistically significant (OR of 0.2321; *p* = 0.19). The ingestion of probiotics also did not show statistical significance in RR, indicating that there was no association between the consumption of probiotics and appearance of initial caries lesions (Table 3), that was confirmed after logistic regression (multivariate analysis) (Table 4 and Table 6).

### 3.6. Effect on Dental Plaque

The groups were not balanced according to the OHI-S scores at baseline with significantly higher value for the control relative to the probiotic group (*p* = 0.04) (Table 1).

Both groups showed a significant decrease in plaque scores between the start of the study and after 2 weeks. In the next time period (2 weeks–3 months), the OHI-S scores in both groups increased (significantly for the control group and insignificantly for the probiotic group), but the decrease in OHI-S scores is still visible for both groups in the time interval between baseline and 3 months after the end of probiotic supplementation, with the difference being statistically significant. In the time interval between 3 and 12 months, the OHI-S scores in both groups increased significantly, which finally resulted in a decrease between the total plaque scores at the start of the study and after 12 months from the end of probiotic supplementation, for both groups, significant (*p* = 0.00) for the control group but nonsignificant for the probiotic group (Table 7).

**Figure 2:**
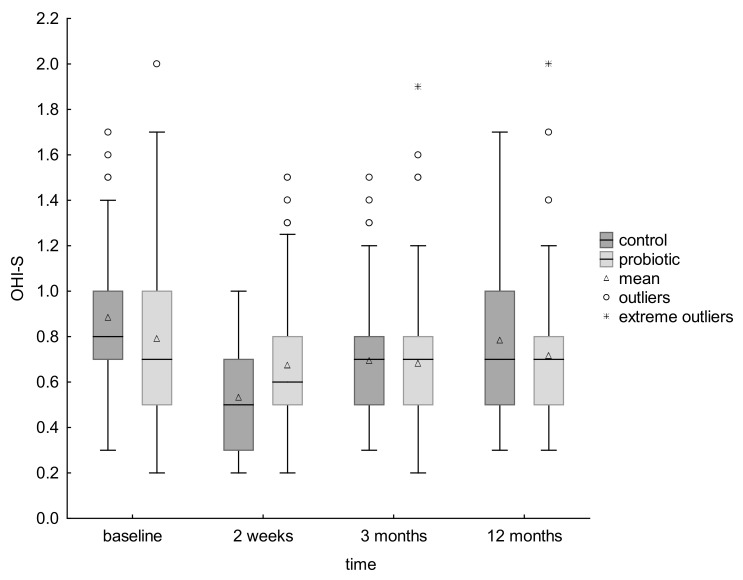
OHI-S (Simplified Oral Hygiene Index) scores at each time point for both groups based on non-parametric Friedman test.

Standard deviation analysis showed that the probiotic group was more diverse in terms of OHI-S at the start of the study and that several children had higher OHI-S values which inflated the mean, and at the end of the study the group was more homogeneous. The values of the coefficient of variation for this group in the next four points of the study were: 0.182; 0.123; 0.118 and 0.0969, respectively. Children who initially showed higher values lowered them, while the rest remained at a comparable level (Table 7) (Figure 2).

No adverse effects were reported by parents during the intervention period.

## 4. Discussion

This clinical trial was performed to test whether the short-term daily consumption of chewing tablets containing thermally inactivated *Lactobacillus salivarius* HM-6 Paradens reduces the caries increment compared with treatment as usual (TAU) control in high-caries preschool children.

The main reason for participants leaving the study was their inability to maintain the pill-taking regime. The observed attrition rate after 12 months at the level of 9.3% seems to correlate with the expected attrition rate (10% per year). However, the observed attrition rate was all for the experimental group which means the actual dropout rate for this group was 18.6%. This high attrition rate could have resulted in a source of bias as individuals who left the study may have different characteristics than those who remained in the study, affecting the homogeneity of the two groups. The baseline general, demographic and clinical characteristics of the children in the intervention group and the control group were compared to assess this issue. The comparison showed no statistically significant differences between the groups and no significant differences in the baseline caries data were found between the children who dropped out and those who attended the final examination (examine groups vs. dropouts). As all the dropouts were from the probiotic group, the children from the probiotic group who continued the study and those who dropped out were also compared, and there were no significant differences between them (probiotic vs. dropouts).

The sample size in the initial power calculation was not fully reached (70 subjects in each arm—sufficient to get significant differences, not enough to cover expected attrition rate), but the caries incidence in the study population was higher than expected which, in part, caused the study not to be underpowered. While the sample size was sufficient at the tooth level, it was relatively small at the patient level, which is the limitation of this trial.

Initially the study was planned as a randomized, triple-blind, placebo-controlled, 2-arm trial, but the prospective investigation was finally a single-center, randomized, controlled open-label, blinded end-point evaluation trial in two parallel groups.

The amendment to the protocol was made at the stage of the pilot study, before the main trial. The reason for the change was the reluctance of guardians, already at the recruitment stage, to participate in a study that required them and their children to maintain a tablet-taking regime, which was difficult due to the fact that the study involved small children, under 6 years of age. In large part, this reluctance was due to the fact that the child might be receiving a placebo and that (according to the parents) there was no additional beneficial effect of the probiotic, while it required a great deal of commitment and effort on their part to give the tablets to their children at a specific time in relation to eating meals and brushing teeth. Many times, children do not eat breakfast until they go to kindergarten, so the tablets would have to be administered by the educators of these institutions, which would not always be possible. The recruitment of children for the study, made more difficult and limited by these objections, prompted the authors to make the above change.

No changes were made to the protocol after the trial commenced.

In the study, it was ensured that all researchers were blinded, while the lack of blindness of the study participants might have caused an error related to the knowledge of the intervention (performance bias). The effect of unblinding in a RCT might lead to a decrease in the response in the control arm and an overestimation of the efficacy in the treatment arm [58]. However, the more objective is the trial’s end point assessed and less reported by the patient (as in the case of the effect of a probiotic), the smaller the need for blinding. Moreover, literature reports suggesting that the widely recognized therapeutic effect of placebo seems to be more pronounced in children than in adults, are noteworthy. This fact, ignored due to contradictory data, together with a lower number of patients per study site, as in our study, associated with a higher placebo response (but not the drug response) could significantly affect the final results, leading in turn to an underestimation of the efficacy in the treatment arm [58,59,60,61].

Additionally, in order to minimize the risk of error in conduct resulting from differences in the care of participants in the compared groups (performance bias), all children participating in this study, from both groups, were included in routine checks as patients of the University Dental Clinic in Kraków. If necessary, dental treatment and professional caries prophylaxis, in accordance with the recommendations as an element of TAU was conducted [54]. Additionally, home caries prophylaxis and dietary recommendations (including compliance with appropriate breaks between meals) were the same in both groups, in line with the existing guidelines [13,15].

The probiotic tablets were sweetened with xylitol, a natural low-cariogenic polyol (200 mg/1 tablet). A 400 mg daily dose of xylitol, based on the existing literature, should not have any effect on the reduction in caries in children. Recent meta-analyses showed low or very low quality of evidence, insufficient to determine whether any xylitol-containing products can prevent caries in children [62,63,64]. Even higher doses of xylitol (>4 g/day) showed only an average reduction in caries but with very low quality of evidence [64].

According to the filled questionnaires and the number of non-used tablets that parents brought back to the clinical team at the end of treatment period, compliance with the study protocol was acceptable. There were no more than two tablets per week forgotten by any participant, with the vast majority of unused lozenges relating to the morning time (87 out of 145 of all missed tablets).

The limitation of this trial was also the short-term nature of the follow-up period. A longer period with more monitoring time-points may be required, in order to assess the durability of the short-time (2-week) probiotic implementation effect in the longer-term follow-up period. Oral examinations in this study were planned to be carried out at baseline and at the end of the study, after 12 months according to time necessary for the development of clinically visible caries lesions [65] and according to the caries management protocol for 3–6-year-old children [66].

Finally, the low incidence of caries at the end of the study in the ICDAS 1–2 range, in both groups, caused values of medians for Δd(ICDAS 1–2) dmft were 0, creating a risk of misinterpretation of the results (Table 2). The distribution of these values may seem were similar if performed solely without looking at the distribution of them. In fact, however, in the probiotic group no new changes appeared, unlike in the control group. The expression of this difference is the value *p* = 0.00244, which shows statistically significant difference in the distributions of values between the groups.

The present study demonstrated a significant reduction in the 1-year increment of early childhood caries incidence and prevalence in high caries rate group of children, following a 2-week daily intake of oral probiotic tablets. Therefore, the null hypothesis was rejected.

Our findings are in accordance with those of Näse et al. (2001), Stecksén-Blicks et al. (2009), Stensson et al. (2014), Hedayati-Hajikand al. (2015), Rodríguez et al. (2016) and Pahumnto et al. (2018); as in all of them, a significant reduction in caries increment or caries risk factors was found [19,20,28,29,30,46].

In contrast, those of Taipale et al. (2013), Hasslöf et al. (2013) and Villaviciencio et al. (2017), did not show a significant caries reduction measured [31,32,34].

Most of the clinical studies with caries as the endpoint support the finding of the present study, that the probiotic bacteria supplement has a certain caries-reducing effect, indicating the potential benefits in ECC prophylaxis. However, it should be emphasized that the direct comparison of the studies is difficult due to a lack of uniformity in design.

To our knowledge, this is the first clinical study that evaluated the effect of a short-term probiotic intervention on the reduction in early childhood caries with 12 months of follow-up. Most of the previous studies on this topic in preschool children have assessed a short-term effect immediately after or up to 3 months after the completion of longer interventions lasting at least 3 months (follow up period = intervention period/+3 months) [19,20,28,29,32,46]. Only the authors of three early (in 1 to 2-year-olds) long-term interventions lasting between 7 and 21 months have studied the long-term effect, over 2–8 years, on the reduction in clinical indicators of caries [30,31,34]. However, those studies were conducted in a low-caries populations, which was in contrast to the current design and may influence the final results due to the fact that probiotic effects are species and/or strain specific [31]. Then, different strains of probiotic bacteria were administered, most often *Lactobacilli* (LBC) [20,28,29,30,34,46] and had no impact on the caries occurrence in only one study [34], but in no study except ours it was *Lactobacillus salivarius*. Sometimes they were also administered in different combinations, with fluoride added, e.g., in the study of Stecksén-Blicks et al. (2009), making it impossible to evaluate the probiotic effect alone [20].

As we know, the environmental conditions should be optimal for the probiotic bacteria to be effective in the oral cavity. The limiting factor may be the short contact time with the oral cavity of externally administered probiotics. Finally, the way of administering the probiotic may be important from the point of view of the results obtained in our study. The use of chewing tablets seems to extend the contact time of probiotic bacteria with the oral cavity compared to milk, most commonly used in previous reports, thus influencing the effectiveness of their action, especially in short-term interventions [19,20,29,32,46].

The only meta-analysis on this topic, taking into account changes in caries parameters as an endpoint, did not show a significant reduction in ECC gain [39], while the systematic reviews available in the literature consistently showed promising research results, indicating a positive effect of probiotics in its prophylaxis [37,40,41,42,47].

According to the secondary outcomes, our results indicated that the probiotic intervention mainly affected the early enamel lesions’ progression rather than appearance of new initial caries lesions, with a relatively small number of children in the test group exhibiting new non-cavitated lesions during the study period. Depending on the severity of caries lesions, the degree of risk of progression varies, which remains in a relationship to the results of research showing that microflora on the tooth surfaces change as caries progresses [67,68]. It is known that the caries lesion can be arrested and even repaired at its initial stages without operative intervention by the net mineral gain improvement during the demineralization and remineralization cycles [65]. This effect can be achieved by reducing the influence of etiological factors such as cariogenic biofilms and diet, and improving the efficacy of remineralizing agents such as saliva and fluoride [69].

In conclusion, the above results may indicate the potentially greater benefits of using *Lactobacillus salivarius* HM-6 Paradens in the secondary prevention of initial caries lesions than in preventing the initiation of the caries process (primary prevention).

In contrast to our results in this matter, Hedayati-Hajikand et al., found that chewing tablets containing three strains of live probiotic bacteria (ProBiora3) mainly affected the appearance of new enamel demineralization rather than progression towards cavitated lesions with no children in the test group exhibiting new caries lesions during the study period [19].

In most cases, the 1-year increment in caries incidence and prevalence was not examined separately for non-cavitated (initial), cavitated and obvious dentinal caries lesion level. Only the results of Villavicienco et al. could be in line with ours regarding the lack of association between the consumption of probiotics and the appearance of initial caries during follow-up, but it also showed no significant differences for both groups compared with respect to total caries increment [32].

In the context of the reduction in caries increment obtained in the probiotic group, the expected result of the study would be a significant decrease in dental plaque accumulation (an established ECC-risk factor) as some previous trials showed [10,11,33,45]. In fact, the initial and final (1-year follow-up) mean OHI-S scores in the probiotic group did not show any significant differences, while the control group noted a significant decrease. The explanation of the obtained reduction in caries increment seems to be the probable decrease in the number of cariogenic microorganisms, including MS, in the plaque with a decrease in its cariogenic potential.

A significant reduction in plaque scores between the start of the study and after 2 weeks for both groups is probably due to the efficient plaque removal carried out not only under the supervision but rather by motivated guardians themselves as a consequence of the dental plaque removing instructions given to all of the participants’ guardians at the beginning of the study. The level of hygiene depends on a large extent of the accuracy of the mechanical removal of plaque during daily tooth brushing procedures and proper oral hygiene is certainly important for biofilm control [70,71,72].

The insignificant plaque score difference between baseline and at the end of the study in the probiotic group may indicate that the regimen implemented during this study was not efficacious for a reduction in dental plaque, as it was shown in some previous trials [19,30,32] and in a meta-analysis by Gruner [39].

However, the effect of the probiotic administered in the first time interval cannot be ruled out, especially considering the statistically significant increase in OHI-S scores visible for the control group in the time interval between 2 weeks and 3 months after the end of probiotic supplementation, while in the test group they remained practically unchanged. It may indicate that the benefit of *Lactobacillus salivarius* HM-6 Paradens treatment extended to 3 months after the termination of the probiotic treatment. A significant reduction in the OHI-S value in the test group during 2-week probiotic supplementation could, therefore, be the result of the combined improvement of hygiene and probiotic action. Additionally, the fact that the positive effect of *Lactobacillus salivarius* HM-6 Paradens treatment did not extend to 12 months after the termination of the probiotic treatment can explain the statistically significant increase in plaque scores between 3 and 12 months and the lack of the significant reduction in the final plaque accumulation, expected in a case of the obtained reduction in 1-year caries increment in the test group.

Finally, it should be noted that only several children in the test group initially had higher OHI-S values, which inflated the mean, and at the end of the study those participants lowered them, while the rest remained at a comparable level as it is probably more difficult to achieve improvement as most children initially had good hygiene. So, there is a possibility that *Lactobacillus salivarius* HM-6 Paradens treatment may provide greater benefit to individuals with high plaque levels at the start.

## 5. Conclusions

Within the limitations, the findings of the present study show that childhood caries increment could be reduced by a 2-week daily intake of chewing tablets containing thermally inactivated *Lactobacillus salivarius* HM-6 Paradens as adjunct to daily use of fluoride toothpaste.

Our findings, obtained 12 months after the end of the intervention, suggest that short-term self-administered probiotic therapy may provide a good complement to increase the effectiveness of the recommended and established individual preventive home care in this age group.

Further studies are needed to confirm these results and to address issues such as dosage, and frequency of delivery before any definitive recommendation as a measure to reduce caries risk can be given. It is not also clear how long the beneficial effects of probiotics last or how long the supplementation must be sustained.

## Figures and Tables

**Table 1:** Baseline characteristics of the children in the probiotic and control groups.

Characteristic	Control Group	Probiotic Group	Dropouts	Control vs. Probiotic	Probiotic vs. Dropouts	Examine Groups vs. Dropouts
**Participants, *n***	70	57	13			
**Male, *n* (%)**	39 (55.71%)	31 (54.39%)	8 (61.54%)	*p* = 0.88 ^b^	*p* = 0.64 ^b^	*p* = 0.66 ^b^
**Female, *n* (%)**	31 (44.29%)	26 (45.61%)	5 (38,.46%)	*p* = 0.88 ^b^	*p* = 0.64 ^b^	*p* = 0.66 ^b^
**Age, mean ± SD**	4.51 ± 0.94	4.59 ± 0.92	4.46 ± 1.45	*p* = 0.76 ^a^	*p* = 0.9 ^a^	*p* = 0.98 ^a^
**ICDAS 1–6 > 0, *n* (%)**	35 (50%)	28 (49.12%)	6 (46.15%)	*p* = 0.93 ^b^	*p* = 0.85 ^b^	*p* = 0.81 ^b^
**d(ICDAS 1–6) mft, mean ± SD**	5.67 ± 6.68	5.93 ± 6.68	5.23 ± 6.29	*p* = 0.83 ^a^	*p* = 0.66 ^a^	*p* = 0.70 ^a^
**OHI-S, mean ± SD**	0.88 ± 0.32	0.79 ± 0.43	0.86 ± 0.48	***p* = 0.04** **^a^**	*p* = 0.72 ^a^	*p* = 0.93 ^a^

^a^ Non-parametric Mann-Whitney (Wilcoxon) test; ^b^ proportion analysis test; SD—standard deviation; ICDAS—International Caries Detection and Assessment System; OHI-S—Simplified Oral Hygiene Index. Boldface indicates statistical significance (*p* < 0.05).

**Table 2:** Dental caries incidence in the probiotic and control group at baseline and end of the study.

Effect	Cohen’s d	Mann-Whitney Test	Q3	Q1	Max	Min	SD	Median	Mean	* n *	Group		
no effect	0.016	*p* = 0.925	9.75	0	20	0	6.68	3	5.671	70	control	**d(ICDAS 1–6) mft, baseline**	
			12	0	20	0	6.68	3	5.93	57	probiotic		
small	0.217	*p* = 0.214	11.75	0	21	0	6.75	6	7.043	70	control	**d(ICDAS 1–6) mft,**	**12 months**
			11	0	20	0	6.26	4	5.825	57	probiotic		
large	1.087	***p* < 0.001**	2	0	7	0	1.63	1	1.371	70	control	**Δ d(ICDAS 1–6) mft,**	**12 months—baseline**
			0	0	4	−6	1.18	0	−0.105	57	probiotic		
no effect	0.074	*p* = 0.661	6	0	14	0	4.38	1	3.429	70	control	**d(ICDAS 1–2) mft, baseline**	
			7	0	15	0	4.75	2	3.947	57	probiotic		
small	0.135	*p* = 0.434	7	0	18	0	5.04	4	4.643	70	control	**d(ICDAS 1–2) mft,**	**12 months**
			8	0	15	0	4.72	3	4.07	57	probiotic		
intermediate	0.517	***p* = 0.002**	3	0	11	−5	2.5	0	1.214	70	control	**Δ d(ICDAS 1–2) mft,**	**12 months—baseline**
			0	0	5	−4	1.45	0	0.123	57	probiotic		
no effect	0.029	*p* = 0.865	7	0	14	0	4.61	3	3.971	70	control	**d(ICDAS 3–6) mft, baseline**	
			7	0	15	0	4.86	2	4.053	57	probiotic		
small	0.215	*p* = 0.218	10	0	21	0	6.35	6	6.5	70	control	**d(ICDAS 3–6) mft,**	**12 months**
			10	0	19	0	5.89	2	5.228	57	probiotic		
intermediate	0.593	***p* < 0.001**	4.75	0	10	−5	2.84	2	2.529	70	control	**Δ d(ICDAS 3–6) mft,**	**12 months—baseline**
			2	0	8	−1	1.99	0	1.175	57	probiotic		
no effect	0.015	*p* = 0.931	5	0	13	0	4.05	1	3.157	70	control	**d(ICDAS 4–6) mft, baseline**	
			6	0	14	0	4.41	1	3.544	57	probiotic		
small	0.278	*p* = 0.11	10	0	21	0	6.29	5.5	6.214	70	control	**d(ICDAS 4–6) mft,**	**12 months**
			8	0	14	0	5.08	2	4.351	57	probiotic		
intermediate	0.697	***p* < 0.001**	6	0	12	−3	3.49	2	3.057	70	control	**Δ d(ICDAS 4–6) mft,**	**12 months—baseline**
			1	0	8	−6	2.01	0	0.807	57	probiotic		

SD—standard deviation; ICDAS—International Caries Detection and Assessment System; Q1—25th percentile; Q3—75th percentile; Cohen’s d—effect size. Boldface indicates statistical significance (*p* < 0.05).

**Table 3:** Dental caries prevalence in the probiotic and control group at baseline and end of the study.

NNT (Harm or Benefit)	Absolute Risk AR (95% CI)	ARR or ARI	Relative Risk RR (95% CI)			Prevented Fraction (PF)	Odds Ratio OR	(95% CI)		Binominal Proportions Test	Control Group		Probiotic Group		
											70		57		*n*
NNT (Benefit) = 114	0.88%	(−16.61%, 18.36%)	0.9825	(0.6902, 1.3985)	*p* = 0.92	3%	0.9655	(0.4797, 1.9432)	*p* = 0.92	*p* = 0.92	35	−50%	28 (49,12%)		**ICDAS 1–6 > 0, baseline, *n* (%)**
NNT (Benefit) = 6.74	14.84%	(−2.25%, 31.93%)	0.7742	(0.5701, 1.0515)	*p* = 0.1013	46%	0.5404	(0.2640, 1.1062)	*p* = 0.092	*p* = 0.09	46 (65.71%)		29 (50.88%)		**ICDAS 1–6 > 0. 12 months. *n* (%)**
NNT (Benefit) = 7.16	13.96%	(4.78%, 23.14%)	0.1116	(0.0149, 0.8391)	***p* = 0.0331**	90%	0.0958	(0.012, 0.7663)	***p* = 0.027**	***p* = 0.075**	11 (15.71%)		1 (1.75%)		**ΔICDAS 1–6 > 0. 12 months—baseline. *n* (%)**
NNT (Harm) = 147.8	0.68%	(−16.57%, 17.92%)	10.163	(0.6727, 1.5355)	*p* = 0.94	0%	1.0282	(0.5060, 2.0892)	*p* = 0.94	*p* = 0.94	29 (41.43%)		24 (42.11%)		**ICDAS 1–2 > 0. baseline. *n* (%)**
NNT (Harm) = 10.53	9.50%	(−5.30%, 24.30%)	15.115	(0.7947, 2.8748)	*p* = 0.21	0%	17.111	(0.7426, 3.9425)	*p* = 0.21	*p* = 0.21	13 (18.57%)		16 (28.07%)		**ICDAS 1–2 > 0. 12 months. *n* (%)**
NNT (Benefit) = 18.56	5.39%	(−1.54%, 12.32%)	0.2456	(0.0295, 2.043)	*p* = 0.19	77%	0.2321	(0.0263, 2.0467)	*p* = 0.19	*p* = 0.15	5 (7.14%)		1 (1.75%)		**ΔICDAS 1–2 > 0. 12 months—baseline. *n* (%)**
NNT (Benefit) = 21.22	4.71%	(−12.70%, 22.12%)	0.9030	(0.6175, 1.3204)	*p* = 0.60	13%	0.872	(0.4098, 1.6698)	*p* = 0.60	*p* = 0.60	34 (48.57%)		25 (43.86%)		**ICDAS 3–6 > 0. baseline. *n* (%)**
NNT (Benefit) = 3.23	30.95%	(14.35%, 47.56%)	0.5185	(0.3453, 0.7786)	***p* = 0.0015**	72%	0.2778	(0.1330, 0.5801)	***p* = 0.0007**	***p* = 0.00052**	45 (64.29%)		19 (33.33%)		**ICDAS 3–6 > 0. 12 months. *n* (%)**
NNT (Benefit) = 4.27	23.43%	(9.34%, 37.53%)	0.3439	(0.1605, 0.7365)	***p* = 0.006**	75%	0.2520	(0.0994, 0.6386)	***p* = 0.0037**	***p* = 0.0025**	25 (35.71%)		7 (12.28%)		**ΔICDAS 3–6 > 0. 12 months—baseline. *n* (%)**
NNT (Benefit) = 7.24	13.81%	(−3.21%, 30.74%)	0.7071	(0.4540, 1.1013)	*p* = 0.12	44%	0.5606	(0.2719, 1.1558)	*p* = 0.12	*p* = 0.12	33 (47,14%)		19 (33.33%)		**ICDAS 4–6 > 0. baseline. *n* (%)**
NNT (Benefit) = 2.67	37.52%	(21.86%, 53.18%)	0.3594	(0.2095, 0.6168)	***p* = 0.0002**	82%	0.1886	(0.0852, 0.4176)	***p* < 0.0001**	***p* = 0.00002**	41 (58.57%)		12 (21.05%)		**ICDAS 4–6 > 0. 12 months. *n* (%)**
NNT (Benefit) = 2.83	35.39%	(23.57%, 47.21%)	0.0472	(0.0066, 0.3375)	***p* = 0.0023**	97%	0.0302	(0.0039, 0.2315)	***p* = 0.0008**	***p* = 0.000001**	26 (37.14%)		1	(1.75%)	**Δ ICDAS 4–6 > 0, 12 months—baseline, *n* (%)**

95% CI—95% confidence interval; ARR—absolute risk reduction; ARI—absolute risk increase; ICDAS—International Caries Detection and Assessment System. Boldface indicates statistical significance (*p* < 0.05).

**Table 4:** Multivariate analysis of Δ ICDAS 3–6 > 0; 12 months—baseline, *n* (%).

Variable	B	Standard Error	*p*	Odds Ratio	95% CI for Odds Ratio	
					Low Limit	Upper Limit
Age	0.176	0.234	0.451	1.193	0.754	1.886
Sex	0.351	0.430	0.415	1.420	0.611	3.299
Group control vs. probiotic	−1.407	0.477	**0.003**	**0.245**	0.096	0.625

B—regression coefficient; 95% CI—95% confidence interval. Boldface indicates statistical significance (*p* < 0.05).

**Table 5:** Multivariate analysis of Δ ICDAS 4–6 > 0; 12 months—baseline, *n* (%).

Variable	B	Standard Error	*p*	Odds Ratio	95% CI for Odds Ratio	
					Low Limit	Upper Limit
Age	0.201	0.263	0.443	1.223	0.731	2.047
Sex	0.270	0.486	0.579	1.310	0.505	3.398
Group control vs. probiotic	−3.533	1.041	**0.001**	**0.029**	0.004	0.225

B—regression coefficient; 95% CI—95% confidence interval. Boldface indicates statistical significance (*p* < 0.05).

**Table 6:** Multivariate analysis of Δ ICDAS 1–2 > 0; 12 months—baseline, *n* (%).

Variable	B	Standard Error	*p*	Odds Ratio	95% CI for Odds Ratio	
					Low Limit	Upper Limit
Age	−0.172	0.450	0.703	0.842	0.349	2.034
Sex	−0.207	0.848	0.807	0.813	0.154	4.282
Group control vs. probiotic	1.453	1.112	**0.191**	**4.276**	0.484	37.786

B—regression coefficient; 95% CI—95% confidence interval. Boldface indicates statistical significance (*p* < 0.05).

**Table 7:** Non-parametric Friedman test of OHI-S (Simplified Oral Hygiene Index).

	OHI-S	*n*	Mean	SD	Median	Q1	Q3	Min	Max	Friedman Test	Durbin-Conover Post Hoc Test ^a^
**Control Group**	Baseline	70	0.88	0.32	0.80	0.7	1.0	0.3	1.7	χ^2^ = 107.833 ***p* = 0.00**	Baseline—2 weeks Baseline—3 months Baseline—12 months 2 weeks—3 months 2 weeks—12 months 3 months—12 months
	2 weeks	70	0.53	0.21	0.50	0.3	0.7	0.20	1.00		
	3 months	70	0.69	0.30	0.70	0.5	0.8	0.30	1.50		
	12 months	70	0.78	0.33	0.70	0.5	1	0.30	1.70		
**Probiotic Group**	Baseline	57	0.79	0.43	0.70	0.5	1	0.2	2.0	χ^2^= 14.9821 ***p* = 0.002**	Baseline—2 weeks Baseline—3 months 3 months—12 months
	2 weeks	57	0.67	0.35	0.60	0.5	0.8	0.20	1.50		
	3 months	57	0.68	0.34	0.70	0.5	0.8	0.20	1.90		
	12 months	57	0.72	0.31	0.70	0.5	0.8	0.30	2.00		

SD—standard deviation; Q1—25th percentile; Q3—75th percentile; ^a^ show statistically significant differences between groups. Boldface indicates statistical significance (*p* < 0.05).

## Data Availability

The data presented in this study are available upon request from the corresponding author. The data are not publicly available due to privacy restrictions.
